# Artificial Pneumothorax—Treatment of Aneurysm—Diathermy

**Published:** 1914-02-07

**Authors:** 


### MODERN METHODS SERIES.—II.

### Artificial Pneumothorax—Treatment of Aneurysm—Diathermy.

Recent reports from various investigators serve to support the claims made for artificial pneumothorax in the treatment of pulmonary tuberculosis. The technique of this method was described at length in The Hospital (March 29, 1913). Dr. Claude Lillingston, of Gorleston-on-Sea, whose name is well known as a pioneer in this field, has lately published an account of his results up to date in the *Clinical Journal* (December 24), and continues to advocate warmly the procedure in certain cases of advanced phthisis, particularly advising it where one lung is very much diseased and the other not yet affected. Dr. S. Vere Pearson, of Mundesley Sanatorium, writes optimistically of results obtained since his interesting address before the British Medical Association at Liverpool (July 1912), when he reported a number of cases in which patients with extensive excavation and caseation of an apparently hopeless kind were greatly benefited by the operation. Dr. Pearson reports that his further experience confirms him in the view that it is a useful method for a few cases; his pneumothorax series showing satisfactory results, such as in his opinion could not have been obtained by any other method. Further recent successes have been reported by Dr. Esther Carling, of Maitland Sanatorium, who has lately published a remarkable instance of restoration to health of a practically moribund case.

Drs. H. de C. Woodcock and J. M. Clark, of Leeds, report that up to the present time they are convinced that pneumothorax often brings a reprieve to a patient suffering from widespread pulmonary tuberculosis. Although it is impossible to say that the patient is cured in successful cases, it is possible to say that the disease has been retarded in its course, limited in its spread, and lightened in its symptoms. These workers consider that great care should be taken to avoid performing the pneumothorax operation in certain conditions which are exceedingly dangerous. They point out that a patient with extensive disease of both lungs, and therefore with a limited working breathing area, can be asphyxiated by the introduction into the pleura of even a moderate amount of compressing gas. A patient in whose pulmonary tissues the circulatory equilibrium is unstable may suffer acute pulmonary œdema as the result of the introduction of a needle into the pleura or lung. This, of course, applies to cases where constant dyspnœa on slight exertion is a symptom of arterio-sclerotic changes. Drs. Woodcock and Clark state that in their experience they have had no fatalities, although in one or two cases they have observed very serious symptoms as a result of the procedure. Thus in one of their cases the patient was intensely ill from shock; in another signs indicative of embolism appeared; and in a third the patient deeply cyanosed, lost consciousness, and became convulsed, the condition remaining acute for several days. They also report that on the other hand they know of many patients now living who otherwise must have succumbed. Thus there appears to be a consensus of opinion amongst those competent to judge that the pneumothorax treatment is most likely to be successful where tubercular infection is unilateral, and that extensive cavitation by itself is no contraindication. It will be remembered that Professor Saugman reported a series of a hundred cases so treated at the International Congress last summer, out of which no less than thirty-two patients were able to resume work.

### Treatment of Aneurysm.

A case brought before the Medical Society of London just before Christmas gave some indication that the treatment for aneurysm known as Colt's method of wiring will be more used in the future, for here the progress of a large saccular dilatation of the aorta was apparently arrested by the process, although the patient soon after succumbed to the consequences of the concomitant heart disease. This method was first introduced some ten years ago, when Mr. G. H. Colt, of Aberdeen University, reported with Mr. D'Arcy Power, of St. Bartholomew's, the case of an abdominal aneurysm treated in this way. The method consists in first of all introducing a trocar and cannula into the sac and, on withdrawal of the latter, introducing a cartridge containing a bundle of closely packed and very fine silver wires which are soldered together in the middle. This having been pushed into the aneurysm cavity the trocar is then withdrawn. Success depends on the expansion of the fine wires in umbrella fashion and their adaptation to the sac without damaging its wall. Coagulation is, of course, relied upon to complete obliteration.

### Diathermy.

Very satisfactory results are reported in connection with the treatment by diathermy, which has lately been on its trial in this country. Briefly, this method consists in applying electrical currents at very high voltage to the human tissues under conditions which obviate danger. Technical details of the diathermy apparatus were described in The Hospital recently (May 13, 1913). According to the size of the applicator used the result obtained is either a coagulation of tissues in its neighbourhood or a general heating of the whole area under treatment, with increased blood supply and loss of sensation to pain. Thus at St. Bartholomew's Hospital the coagulatory effect is being used with excellent results in the treatment of inoperable malignant growths, as well as for rodent ulcer and advanced lupus. Under these circumstances an anæsthetic is always given for the application, during which the current is gradually increased until the tissues around the smaller applicator become colourless. At the same time the general heating effect, secured by means of large metal applicators with which it is not found difficult to heat the whole or part of the limb, is being found of great assistance in the treatment of sciatica, severe metatarsalgia, and chronic arthritis.

